# Development and characterization of a novel model of invasive pneumococcal pneumonia in invasively ventilated PIGS

**DOI:** 10.1186/2197-425X-3-S1-A619

**Published:** 2015-10-01

**Authors:** G Li Bassi, R Amaro, C Chiurazzi, E Aguilera Xiol, C Travierso, L Fernandez Barat, A Motos, M Schultz, M Carbonara, M Rigol, D Marti, M Saco, T Comaru, J Ramirez, A Torres

**Affiliations:** Hospital Clinic de Barcelona, Pulmonary and Critical Care Medicine, Barcelona, Spain; University of Milano. Fondazione IRCCS Ca' Granda Ospedale Maggiore Policlinico, Milan, Italy; Academic Medical Center, University of Amsterdam, Amsterdam, Netherlands; Hospital Clinic de Barcelona, Pathology, Barcelona Spain; Pontifícia Universidade Catolica, CNPq fellow (2012-2014), Rio Grande do Sul, Brazil

## Introduction

*Streptococcus pneumoniae* is the most common causative pathogen of community-acquired pneumonia (CAP). Often, patients with pneumococcal CAP are admitted into intensive care units, particularly when affected by invasive serotypes.

## Objectives

We aimed to develop and characterize a novel model of invasive pneumococcal pneumonia in pigs on mechanical ventilation.

## Methods

Eight female Large White-Landrace pigs (33.9 ± 2.1 Kg) were anesthetized, tracheally intubated and connected to a mechanical ventilator. Ultrasound-guided arterial cannulation was performed, and a Swan-Ganz catheter was inserted into the jugular vein for hemodynamic monitoring. Following surgical preparation, and 4 hours thereafter, 15 mL of 10^8^ cfu/mL of *S. pneumonia* serotype 19A, resistant to erythromycin, penicillin and tetracyclins, was instilled into each pulmonary lobe using a bronchoscope. Throughout the study, quantitative cultures of broncho-alveolar lavage (BAL) and blood cultures were performed. Every 24 hours, gas exchanges, pulmonary mechanics, hemodynamics and laboratory parameters were assessed. Fluids and vasoactive drugs were administered to sustain hemodynamic stability. Doxycyclin and aztreonam were administered to avert colonization by endogenous pathogens. The animals were euthanized 72 hours after bacterial inoculation and pulmonary lobes were sampled for quantitative microbiology studies. Pneumonia was confirmed if pulmonary tissue *S. pneumoniae* culture was ≥ 3 log CFU/gram tissue. Additionally, in 6 animals lung histology was evaluated.

## Results

Five out of eight animals completed the study. Three animals were euthanized earlier, due to pulmonary/hemodynamic failure, unresponsive to treatment. Clinical data are reported in table 1. Vasoactive drugs were used in 5 out of 8 animals. BAL *S. pneumoniae* concentration was 0, 5.9 ± 0.9, 3.6 ± 2.2 CFU/mL, at baseline, 24 and 72 hours of MV, respectively (p < 0.001). *S. pneumoniae* bacteremia was found in 3 animals. Pneumococcal pneumonia developed in 6 out of 8 animals and it was histologically confirmed in all analyzed samples. *S. pneumoniae* concentration of the right upper, medium, lower lobes and the left upper and lower lobes was 4.8 ± 2.3, 4.6 ± 3.1, 3.5 ± 3.2, 3.9 ± 2.9 and 3.4 ± 3.5 CFU/gram, respectively (p = 0.848).

**Table 1:** Clinical data throughout study time.

	Before Bacterial Challenge	24 hours	48 hours	72 hours	P-value
Body Temperature (°C)	36.8 ± 0.9	38.7 ± 0.6	38.6 ± 0.8	38.6 ± 0.5	< 0.001
(°C) White Blood Cell (cells/µL)	11,833 ± 4,706	8,237 ± 2,443	19,070 ± 9,229	14,817 ± 7,579	0.042
Platelets (cells/µL)	423,125 ± 104,120	228,625 ± 60,133	164,712 ± 65,953	209,000 ± 71,288	< 0.001
PaO2/FiO2	441.7 ± 62.7	321.1 ± 35.7	288.3 ± 89.9	375.6 ± 52.6	< 0.001
PaCO2 (mmHg)	47.2 ± 6.3	56.0 ± 8.6	41.0 ± 6.0	37.0 ± 5.7	< 0.001
Lung Elastance (cmH2O/L)	14.2 ± 3.2	28.6 ± 16.1	26.5 ± 15.7	26.6 ± 0.5	0.301
Pulmonary Shunt (%)	4.2 ± 2.0	14.8 ± 4.9	13.5 ± 2.9	8.9 ± 3.2	< 0.001
Mean Arterial Pressure (mmHg)	81.2 ± 10.4	68.3 ± 5.4	68.4 ± 3.9	72.9 ± 8.4	0.074
Systemic Vascular Resistance (dynes*sec/cm5)	1857 ± 643	1048 ± 355	1142 ± 377	1419 ± 516	0.014

## Conclusions

We developed a new model of severe invasive pneumococcal pneumonia characterized by respiratory and hemodynamic failure, hematologic changes and coagulopathy. The model could be used to study the pathophysiology of the disease and novel therapeutic and preventive strategies.

